# Sphingomyelin Functions as a Novel Receptor for *Helicobacter pylori* VacA

**DOI:** 10.1371/journal.ppat.1000073

**Published:** 2008-05-23

**Authors:** Vijay R. Gupta, Hetal K. Patel, Sean S. Kostolansky, Roberto A. Ballivian, Joseph Eichberg, Steven R. Blanke

**Affiliations:** 1 Department of Microbiology, Institute for Genomic Biology, University of Illinois, Urbana, Illinois, United States of America; 2 Department of Biology and Biochemistry, University of Houston, Houston, Texas, United States of America; University of California San Diego, United States of America

## Abstract

The vacuolating cytotoxin (VacA) of the gastric pathogen *Helicobacter pylori* binds and enters epithelial cells, ultimately resulting in cellular vacuolation. Several host factors have been reported to be important for VacA function, but none of these have been demonstrated to be essential for toxin binding to the plasma membrane. Thus, the identity of cell surface receptors critical for both toxin binding and function has remained elusive. Here, we identify VacA as the first bacterial virulence factor that exploits the important plasma membrane sphingolipid, sphingomyelin (SM), as a cellular receptor. Depletion of plasma membrane SM with sphingomyelinase inhibited VacA-mediated vacuolation and significantly reduced the sensitivity of HeLa cells, as well as several other cell lines, to VacA. Further analysis revealed that SM is critical for VacA interactions with the plasma membrane. Restoring plasma membrane SM in cells previously depleted of SM was sufficient to rescue both toxin vacuolation activity and plasma membrane binding. VacA association with detergent-resistant membranes was inhibited in cells pretreated with SMase C, indicating the importance of SM for VacA association with lipid raft microdomains. Finally, VacA bound to SM in an *in vitro* ELISA assay in a manner competitively inhibited by lysenin, a known SM-binding protein. Our results suggest a model where VacA may exploit the capacity of SM to preferentially partition into lipid rafts in order to access the raft-associated cellular machinery previously shown to be required for toxin entry into host cells.

## Author Summary

Sensitivity to toxins produced by pathogenic bacteria is largely dictated by the presence or absence of toxin receptors on the plasma membrane of host cells. VacA is an important toxin produced by the pathogenic bacterium *Helicobacter pylori*, which infects the human stomach and causes gastric ulcer disease and stomach cancer. VacA binds and enters human cells, and induces several changes resulting ultimately in the death of the intoxicated cells. However, the identity of the VacA receptor responsible for toxin binding and function has remained a topic of debate. In this paper, we demonstrate that sphingomyelin, a lipid on the surface of cells with important membrane structural and signaling properties, functions as a VacA receptor. We demonstrate that VacA binds to sphingomyelin, and that presence or absence of sphingomyelin on the plasma membrane dictates how much VacA binds to the cell surface, and therefore, how sensitive cells are to the toxin. The identification of sphingomyelin also provides a conceptual framework for how VacA may enter cells through specialized functional domains on the surface of cells. This is the first example of a bacterial toxin that exploits sphingomyelin as a receptor, and future work will focus on developing strategies to block VacA interactions with sphingomyelin, thereby protecting cells from the downstream consequences of toxin action.

## Introduction

The vacuolating cytotoxin (VacA) is an intracellular-acting toxin generated by the pathogen, *Helicobacter pylori*, which infects the gastric epithelium of humans and is a significant risk factor for the development of peptic ulcer disease, distal gastric adenocarcinoma, and gastric lymphoma in humans [1]. VacA has been demonstrated to be important for *H. pylori* colonization and disease pathogenesis [2]–[5]. Intoxication with VacA results in multiple consequences, including vacuolation and apoptosis of epithelial cells [6].

Analogous to many other bacterial toxins [7], VacA interacts with the plasma membrane of sensitive cells as the first step during intoxication [8]. Subsequent to binding, VacA is internalized by a novel pinocytic-like mechanism, and functions from an intracellular compartment [8]-[13]. There has been considerable interest in identifying target cell receptors for VacA because, for most toxins, the presence or absence of specific receptors on the surface of target cells largely dictates cellular tropism [14]. Several studies have characterized VacA binding to the plasma membrane of sensitive cells as non-specific [10],[15], suggesting that VacA may bind to multiple receptors and/or a highly abundant membrane component. Receptor protein tyrosine phosphatases (RPTP), α and β, have been demonstrated to confer cellular sensitivity to VacA and directly interact with VacA [16]-[18], but the importance of RPTP-α or RPTP-β for binding VacA to the plasma membrane surface has not been demonstrated, and the role, if any, of these proteins as primary binding determinants for VacA is uncertain. VacA interacts *in vitro* with heparan sulfate [19] and several glycosphingolipids [20], but neither the interaction of VacA with these molecules on the surface of mammalian cells, nor the importance of these potential interactions for toxin function has been demonstrated. Likewise, although VacA interacts with lipid preparations and binds to artificial membranes [21]–[26], the specificity and importance of VacA-lipid interactions for toxin function remains to be established. Finally, VacA association with lipid rafts has been demonstrated to be important for the toxin's vacuolation activity [21], [27]–[29], but it is not clear whether VacA binding to lipid or protein components within these rafts is important for toxin function. Thus, the molecular basis of VacA interactions with the plasma membrane and, in particular, the identity of receptors important for VacA binding, remains poorly understood.

To provide further insight into the molecular basis underlying the capacity of VacA to bind to the surface of epithelial cells and induce vacuolation activity, we evaluated the importance of several common membrane lipids for toxin function. Here, we provide evidence that the membrane lipid, sphingomyelin (SM), comprising sphingosine, a fatty acid, a phosphate group, and choline, modulates the sensitivity of epithelial cells to VacA. SM is a key structural component of the cell membrane and, in particular, specialized membrane domains called lipid rafts, while at the same time serving a significant functional role, as it is the parent compound of several lipid mediators [30]. We demonstrate that SM is important for VacA binding to cells and association with lipid rafts on the plasma membrane. Finally, VacA binds *in vitro* to SM in a manner that is competitively inhibited by the SM-specific binding protein, lysenin. These results suggest that SM functions as a receptor for VacA by promoting association of the toxin with plasma membrane lipid rafts.

## Results

### Plasma Membrane SM Confers Cell Sensitivity to VacA

**Figure 1:**
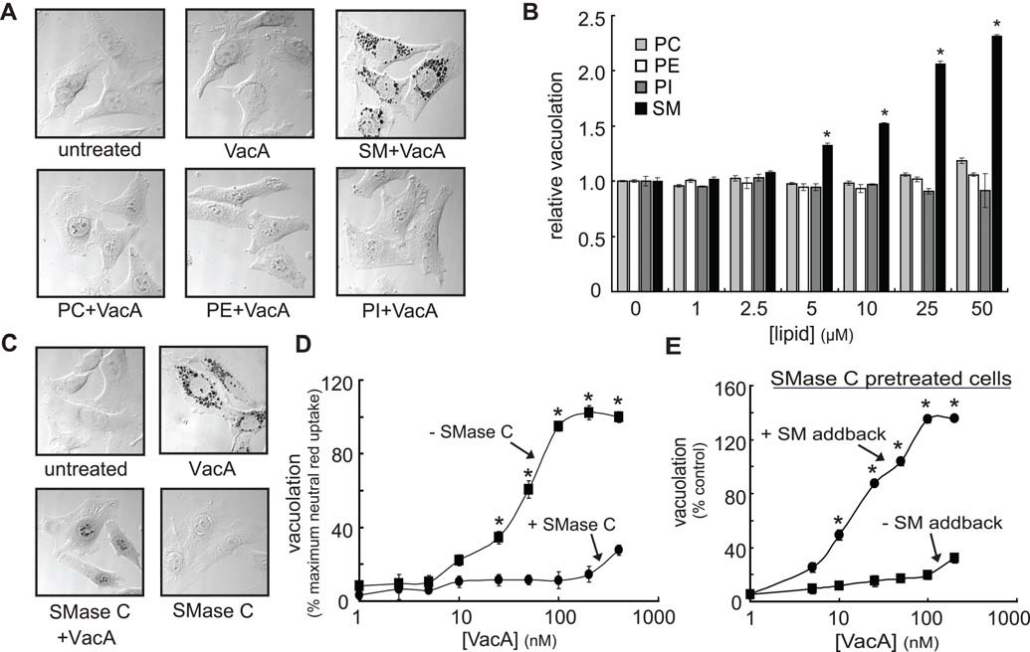
VacA-Mediated Vacuolation is Sensitive to Supplementation or Depletion of Cellular SM. HeLa cells were pretreated for 1 h with 50 μM (A) or 0–50 μM (B) PC, PE, PI, or SM, 100 mU/mL SMase C (C–E), or mock-pretreated with PBS pH 7.2 (A–E). The monolayers were then further incubated at 37 °C with 10 nM (A–B), 100 nM (C), or 0–400 nM (D) VacA, or, supplemented with exogenous SM (50 μM) for 1 h prior to incubation at 37 °C with 0–200 nM VacA (E). After 24 h, cells were evaluated by DIC microscopy (A,C) or neutral red uptake assays (B,D,E). (A) DIC images of cells stained with neutral red. (B) Relative vacuolation of cells preincubated with or without lipids. Vacuolation data were normalized for neutral red uptake in mock-pretreated cells incubated with VacA. Asterisks indicate statistically significant differences in neutral red uptake between cells pretreated with SM and mock-pretreated cells. (C) DIC images of cells stained with neutral red. (D) Relative vacuolation of cells preincubated with or without SMase C prior to toxin exposure. The data were normalized for the highest level of neutral red uptake measured within these experiments. Asterisks indicate statistically significant differences in neutral red uptake between cells pretreated with SMase C and mock-pretreated cells. (E) Relative vacuolation of cells preincubated with SMase C, and then incubated with or without SM prior to toxin exposure. The data were normalized for neutral red uptake of mock-pretreated incubated with VacA. Asterisks indicate statistically significant differences in neutral red uptake between cells with and without SM add-back.

Monolayers of HeLa cells were preincubated at 4 °C with 50 μM exogenous SM, PC, PI, or PE in order to increase the concentration of these selected lipids, which are commonly found in the plasma membrane of mammalian cells. After 1 h, VacA was added to the monolayers at a low concentration (10 nM), and the cells were further incubated with both toxin and exogenous lipids at 37 °C. After 24 h, only modest levels of vacuolation were visible in the absence of exogenous lipids (Figure 1A). However, cellular vacuolation was visibly increased only in monolayers that had been preincubated with exogenous SM, but not in monolayers that had been preincubated with the other lipids (Figure 1A). Essentially identical results were obtained, regardless of whether monolayers were preincubated with SM for 1 h prior to toxin addition, or whether mixtures of VacA and SM were added simultaneously to cells (Figure S1). None of the lipids alone induced vacuolation (data not shown). Notably, the SM content of cells (Table S1) as well as vacuolation (Figure 1B) increased as a function of exogenous SM concentration.

SM is an important and abundant sphingolipid on the plasma membrane of many mammalian cells [31]. To evaluate the effects of reducing cellular SM on VacA-mediated vacuolation, HeLa cells were preincubated for 1 h with SMase C, a phosphodiesterase that hydrolyzes plasma membrane SM to ceramide and free phosphorylcholine at the aqueous-lipid interface, and then further incubated for 24 h with a higher concentration of VacA (100 nM). SMase was previously used to demonstrate binding to SM on the surface of cells by lysenin, the first identified SM-binding protein originally discovered within the coelomic fluid of the earthworm *Eisenia foetida* [32],[33]. Pretreatment of cells with SMase C visibly reduced VacA-mediated cellular vacuolation (Figure 1C). Moreover, SMase C pretreatment visibly reduced plasma membrane SM, as indicated by the reduced fluorescence associated with cells stained with Venus-lysenin (Figure S2). Cellular vacuolation (Figure S3) and SM content (Table S2) were both quantitatively reduced as a function of SMase C concentration. VacA-mediated cellular vacuolation and cellular SM levels were also reduced when cells were pretreated with inhibitors of *de novo* sphingolipid biosynthesis, Fumonisin B_1_ (50 μg/mL) or PDMP (50 μM) (data not shown). However, in subsequent experiments we used SMase C to deplete cellular SM, because of the high level of specificity of SMase C for SM [34],[35], as well as the capacity to specifically hydrolyze SM on the plasma membrane surface.

In addition to HeLa cells, we found that SM was critical for VacA-mediated vacuolation of AGS and AZ-521 cells, two human gastric epithelial cell lines (Figure S3), as well as for CHO-K1 and Vero cells (data not shown), demonstrating that the importance of plasma membrane SM for toxin function is not idiosyncratic to HeLa cells. In subsequent experiments, we chose to use HeLa cells because they have been the most widely used model to investigate VacA association with and entry into host cells [8], [10]–[13],[15],[29],[36]. However, during the course of these studies, we repeated each of the experiments at least once using AZ-521 cells, which yielded essentially identical results to those obtained for experiments employing HeLa cells.

Significantly higher VacA concentrations were required to induce detectable vacuolation in cells preincubated with SMase C relative to untreated cells (Figure 1D); at the highest toxin concentration tested (400 nM), the level of vacuolation was approximately only 30% of that detected for cells in which SM had not been depleted. Furthermore, adding exogenous SM (50 μM) back to cells that had been preincubated with SMase restored the sensitivity of both HeLa (Figure 1E) and AZ-521 cells (data not shown) to VacA. In contrast, adding exogenous PC, PE, PI, or cholesterol back to HeLa cells that had been preincubated with SMase did not restore cellular sensitivity to VacA, even at very high concentrations of lipids (200 μM) (Figure S4). These results demonstrate the importance of plasma membrane SM for conferring sensitivity to VacA.

### Ceramide Enrichment or PC Cleavage is not Inhibitory for VacA-Mediated Cellular Vacuolation

**Figure 2:**
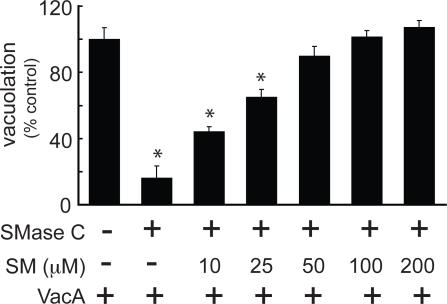
Exogenous SM is Sufficient to Restore Cell Line Sensitivity to VacA. HeLa cells were pretreated with SMase C (100 mU/mL) or mock-pretreated with PBS pH 7.2 for 1 h, washed, and then further incubated with SM (0–200 μM). After 1 h, the cells were washed and incubated with VacA (100 nM), and scored for neutral red uptake after 24 h. The data were normalized to the mock-pretreated cells incubated with VacA. Asterisks indicate statistically significant differences in neutral red uptake between cells pretreated with SMase C and mock-pretreated cells.

We next evaluated whether the inhibitory effects of SMase C were primarily attributable to the loss of SM or, alternatively, to the generation of ceramide, which is a potent mediator of cellular signaling [37]–[39]. Preincubation of HeLa cells with exogenous ceramide had no effect on VacA-mediated vacuolation (Figure S5A). However, VacA-mediated vacuolation was inhibited in a dose-dependent fashion when HeLa cells were preincubated with SMase D (Figure S5B), a phosphodiesterase that cleaves SM at the choline-phosphate linkage to yield ceramide-1-phosphate [34],[40],[41], which has a different and distinct role from ceramide in cell signaling [42],[43]. Finally, adding exogenous SM back to cells that had been preincubated with SMase C restored sensitivity of these monolayers to VacA in a manner that was dependent on the concentration of exogenous SM (Figure 2). These results are consistent with the idea that the inhibitory action of SMase C is primarily due to the loss of plasma membrane SM rather than the generation of ceramide, and further illustrate that the presence of SM at the plasma membrane is important for VacA cellular intoxication.

We also evaluated whether contaminating phospholipase C (PLC) activity within our SMase preparations might contribute to the inhibition of vacuolation. PC-PLC cleaves PC into phosphorylcholine and diacylglycerol (DAG), which is a potent activator of protein kinase C (PKC) signaling). For these studies, we purchased SMase C that had undergone quality control testing to ensure the presence of minimal contaminating PLC activity. Nonetheless, we directly tested and found that neither SMase C nor SMase D induced detectable decreases in cellular PC content (Table S3). Moreover, we determined that SMase C pretreatment inhibited VacA-mediated cellular vacuolation to the same extent in the presence or absence of the PKC inhibitor, BIM I (0 – 5 μM; data not shown), indicating that PKC activation due to PC cleavage was not likely to be responsible for the inhibition of cellular vacuolation. Taken together, these results further support the model that SM depletion, and not low levels of PC cleavage, is responsible for the inhibitory action of SMase C.

### SM is Important for an Early VacA Intoxication Step

**Figure 3:**
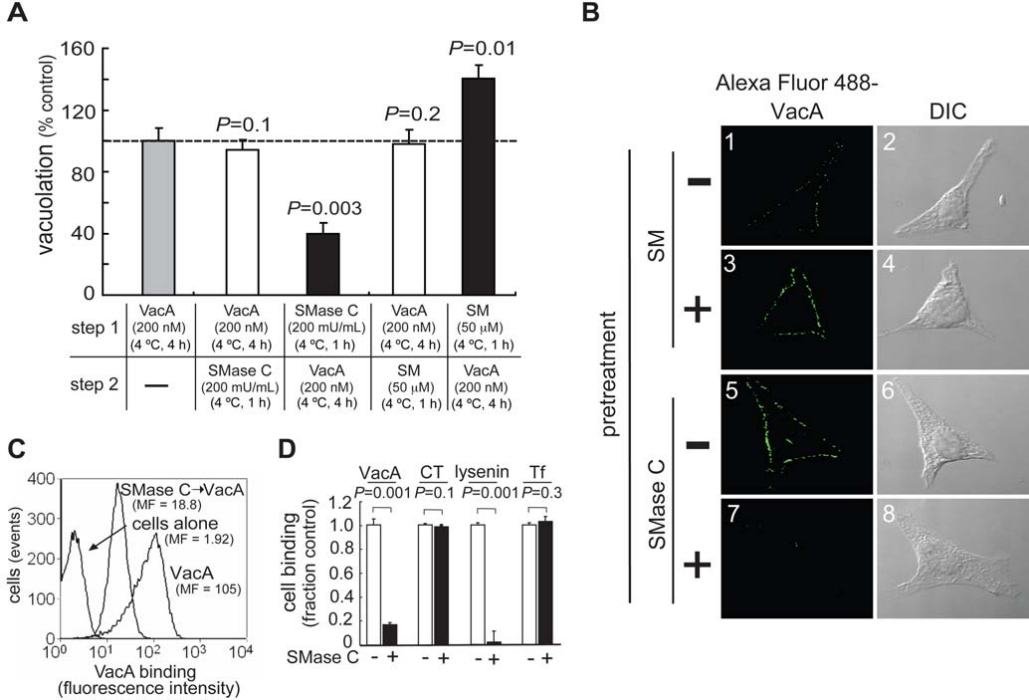
SM is Important for VacA Cell Binding. (A) HeLa cells were treated in two different ways. First, cells were incubated for 1 h with SMase C (200 mU/mL; black bar), SM (50 μM; black bar), or mock-pretreated with PBS pH 7.2 (shaded bar) as indicated. The monolayers were then incubated for 4 h with VacA (200 nM) at 4 °C, and washed with PBS. Alternatively, cells were first incubated for 4 h with VacA (200 nM) at 4 °C and then incubated for 1 h with SM (50 μM; white bar) or SMase C (200 mU/mL; white bar), as indicated, and washed with PBS. For both cases, the monolayers were then incubated for an additional 24 h at 37 °C, and vacuolation was evaluated by measuring neutral red uptake. (B–D) HeLa cells were pretreated for 1 h with or without SM (50 μM) (B, panels 1–4) or SMase C (100 mU/mL) (B, panels 5–8; C–D). The monolayers were then incubated at 4 °C with 10 nM (B, panels 1–4) or 100 nM (B, panels 5–8; C–D), respectively, of Alexa Fluor 488 labeled VacA. For (D), cells were also incubated with Alexa Fluor 488-labeled cholera toxin B fragment (CTB) (100 nM), Alexa Fluor 488-labeled transferrin (60 nM), or Venus-lysenin (1 μM). After 1 h, the cells were analyzed by DIC/epifluorescence microscopy (B) or flow cytometry (C, D). For (C), MF is the geometric mean fluorescence. (A) VacA-mediated vacuolation in cells treated with SM or SMase C, as indicated, before (black bars) or after (white bars) exposure to toxin, relative to non-pretreated cells (shaded bar) exposed to VacA. Vacuolation data were normalized for neutral red uptake in mock-pretreated cells incubated with VacA. *P* values were calculated to determine statistically significant differences between cells pretreated with SM or SMase C and mock-pretreated cells. (B) Fluorescence micrographs (left-hand panels) and DIC images (right-hand panels) of VacA binding to cells that had been pretreated with (panels 3–4) or without (panels 1–2) SM, or, with (panels 7–8) or without SMase (panels 5–6). (C) Flow cytometry histograms of cells alone, cells incubated with VacA, and cells that had been pretreated with SMase prior to exposure to VacA, as indicated. (D) Relative binding of VacA, CTB, Venus-lysenin, and Tf, as indicated, to cells that had been preincubated with (black bars) or without (white bars) SMase. Binding data were normalized for geometric mean fluorescence of each fluorescently-labeled protein bound to mock-pretreated cells. For each protein, *P* values were calculated to determine statistically significant differences in cell binding between cells pretreated with SMase C and mock-pretreated cells.

In the studies described above, VacA-induced vacuolation was modulated in response to preincubating monolayers with exogenous SM or SMase C. To probe the functional role of SM for VacA cellular intoxication, HeLa cells were first incubated with VacA at 4 °C, which promotes toxin binding to the plasma membrane while preventing internalization [10]. The cells were then incubated with exogenous SM or SMase C to increase or reduce plasma membrane SM, respectively. Finally, the cells were incubated at 37 °C for 24 h, and evaluated for cellular vacuolation by measuring neutral red uptake. In contrast to experiments in which cells were preincubated with exogenous SM or SMase (Figure 1 and 3A), vacuolation was not altered when cells with pre-bound VacA were subsequently treated with SM or SMase C (Figure 3A). One interpretation of these data is that pre-bound VacA blocks SMase access to its substrate on the surface of cells. Alternatively, these results may indicate that only the first step along the VacA intoxication pathway – toxin binding to the plasma membrane surface - is sensitive to depletion or supplementation of plasma membrane SM. In either case, these results suggest that plasma membrane SM may be important for toxin association with the plasma membrane surface.

### Plasma Membrane SM is Important for VacA Binding to Cells

We next evaluated the importance of SM for the binding of VacA to the surface of sensitive cells. DIC/fluorescence microscopy revealed that relative to untreated cells, visibly more Alexa Fluor 488-labeled VacA associated with the plasma membrane of HeLa cells preincubated with exogenous SM, while considerably less toxin was visible on the surface of cells pretreated with SMase C (Figure 3B). Flow cytometry measurements revealed that depletion of plasma membrane SM reduced VacA binding by more than 80% (Figure 3C and 3D), while not affecting the binding of transferrin (Tf) or the cholera toxin B fragment (CTB) (Figure 3D). As a control, we demonstrated that pretreatment of cells with SMase C also significantly reduced binding of Venus-lysenin (Figure 3D). Finally, we demonstrated that the level of VacA binding was strongly dependent (R^2^ = 0.95) on the levels of SM in cells pretreated with different concentrations of SMase C (Figure S6).

### Exogenous SM Rescues VacA Binding to SM-Depleted Cells

**Figure 4:**
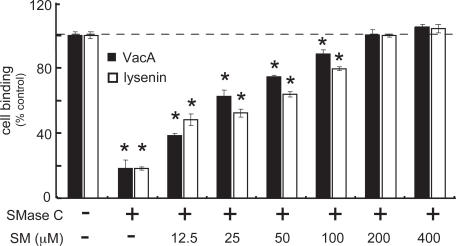
Exogenous SM Rescues VacA Binding to SM-Depleted Cells. Monolayers of HeLa cells were pretreated with SMase C (100 mU/mL) or mock-pretreated with PBS pH 7.2 at 37 °C and under 5% CO_2_. After 1 h, the cells were washed, chilled to 0 °C on ice, and incubated for 1 h with exogenous SM at the indicated concentrations. The cells were then incubated with Alexa Fluor 488-labeled VacA (10 nM) or lysenin-GFP (1 μM) at 4 °C. After 1 h, protein binding was quantified by flow cytometry. Cell binding was normalized to the mean geometric fluorescence of Alexa Fluor 488-labeled VacA binding to mock-pretreated cells. Asterisks indicate statistically significant differences in VacA binding to cells treated with SMase C and mock-pretreated cells.

To further evaluate the importance of plasma membrane SM for VacA interactions with the cell surface, we next determined whether restoring plasma membrane SM back to cells that had been previously depleted of SM was sufficient to rescue VacA binding. As expected, VacA binding was significantly decreased by pretreatment of cells with SMase C (Figure 4). However, when these SM-depleted cells were incubated with exogenous SM prior to exposure to toxin (all at 4 °C), VacA binding increased as a function of SM concentration, and was fully restored at 200 μM SM to levels measured in controls using untreated cells (Figure 4). Similar results were obtained when these experiments were conducted with Venus-lysenin, which was used a control because this protein demonstrates absolute binding specificity for SM on the surface of cells (Figure 4). The increase in cellular binding of Venus-lysenin as a function of SM concentration also supports the premise that exogenously added SM properly inserted into the plasma membrane. In contrast to those results obtained with SM, VacA cellular binding was not restored in the presence of exogenous PC, PE, PI, or cholesterol, even at high concentrations of exogenous lipids (200 μM) (Figure S7). Thus, restoring plasma membrane SM on cells previously depleted of plasma membrane SM was sufficient to rescue binding of VacA to the cell surface.

### VacA Association with Detergent Resistant Membranes (DRMs) Requires SM

**Figure 5:**
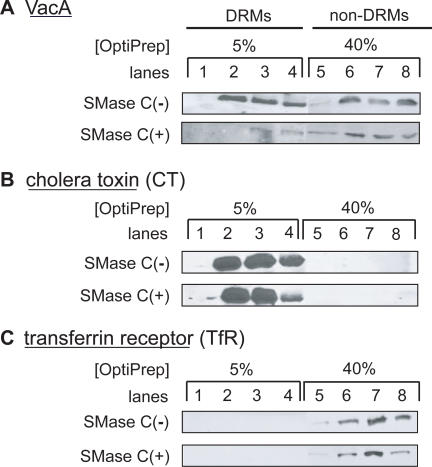
VacA Association with Lipid Rafts Requires SM. HeLa cells (5×10^6^ cells) were preincubated with (+) or without (−) SMase C prior to incubation with VacA (500 nM), CT (500 nM), or mock-incubation with PBS pH 7.2 at 4 °C. After 1 h, the membranes were extracted with ice-cold Triton X-100, and the soluble and insoluble fractions were separated using OptiPrep density gradient centrifugation. Fractions were collected and fractionated by SDS-PAGE. The samples in each lane were normalized according to fraction volumes. VacA (A), CT (B), or Tf-R (C) was visualized by Western blot analysis. Blots from cells pretreated with SMase C or mock-pretreated with PBS pH 7.2 were incubated together with primary and secondary antibodies, and were developed in the same autoradiography cassette for 15 min. Rafts are located within the 5% OptiPrep gradient fractions (lanes 1–4), while non-raft fractions are located within the 40% OptiPrep gradient fractions (lanes 5–8). (A) Western blot analysis of OptiPrep fractions showing the distribution of cross-reacting material to VacA rabbit antiserum. (B) Western blot analysis of OptiPrep fractions showing the distribution of cross-reacting material to anti-cholera rabbit antiserum. (C) Western blot analysis of OptiPrep fractions showing the distribution of cross-reacting material to anti-Tf-R mouse antibodies.

VacA association with lipid raft microdomains on the plasma membrane of mammalian cells is important for the toxin's vacuolating activity [27]–[29]. To explore whether SM is an important determinant for VacA association with lipid rafts, we used gradient density centrifugation to fractionate DRMs, which are the biochemical correlate of lipid rafts, that had been extracted from cells incubated with VacA, cholera toxin (CT), or mock treated with PBS pH 7.2 at 4 °C. As previously reported [15],[27],[29], most of the cell-associated VacA was found in low density fractions, although VacA was also detected within the less buoyant fractions near the bottom of the gradient (Figure 5A). Analysis of DRMs from VacA-treated cells that had been pretreated with SMase C revealed that VacA was visibly reduced in the low-density fractions compared with cells that had not been pretreated with SMase C, but only modestly decreased within the less buoyant fractions near the bottom of the gradient (Figure 5A). In contrast, depletion of cellular SM with SMase C had no effect on the total cellular binding and distribution within the density gradient fractions of either CT (Figure 5B), in agreement with a recent report [44], or the endogenous transferrin receptor (Tf-R) (Figure 5C). These results distinguish VacA- and CT-populated DRMs by their sensitivity to SM depletion, and indicate the importance of SM for VacA association within lipid rafts.

### VacA Binds to SM *in vitro*

**Figure 6:**
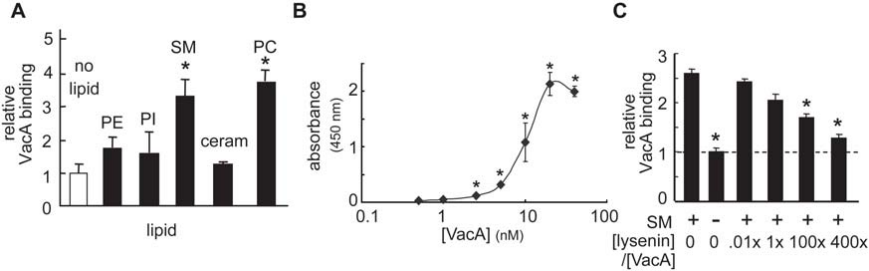
VacA Binds to SM *in vitro.* VacA (10 nM, A and C; or, 0–40 nM, B) was incubated in 96-well Immulon 1B microtiter plates, with individual wells coated with the indicated lipid (500 pmol) in the absence (A,B) or presence (C) of Venus-lysenin. After 2 h, unbound VacA was washed out of the wells, and the extent of VacA binding was measured by ELISA. *P* values were calculated for differences in binding in the presence and absence of lipid (A,B) or lipid plus Venus-lysenin (C). (A) Relative VacA binding to the indicated lipids. Relative VacA binding was calculated by dividing the absorbance at 450 nm in the presence of lipid by that detected in the absence of lipid. Asterisks indicate significant differences between VacA binding to wells containing or not containing lipid. (B) VacA binding to SM as a function of toxin concentration. The background absorbance (VacA binding in the absence of lipid) was subtracted from the absorbance in the presence of SM. Asterisks indicate significant differences in absorbance from wells containing or not containing SM. (C) VacA binding to SM in the presence of different concentrations of Venus-lysenin, relative to VacA binding in the absence of lipid. Asterisks indicate significant differences between VacA binding to SM-coated wells in the absence of lysenin and all other wells.

We next explored the possibility that VacA binds directly to SM, using an ELISA-based assay which has been extensively used for characterizing the binding of lysenin to SM [32],[45],[46]. Significantly more VacA was bound to wells coated with SM than to wells with PE, PI, or in wells not coated with lipids (Figure 6A), similar to what was previously reported for lysenin [33]. VacA binding to SM was dose-dependent, saturable, and detectable at concentrations as low as 2.5 nM VacA (Figure 6B). Consistent with previous reports [10],[15], VacA binding to HeLa or AZ-521 cells was not saturable and only slightly inhibited (<20%) in the presence of a molar excess of unlabeled VacA (data not shown), which we speculate may be explained, at least in part, by the high abundance of SM in the outer leaflet plasma membrane.

Notably, VacA did not bind to ceramide (Figure 6A), which is a product of SMase C-mediated SM hydrolysis lacking the phosphorylcholine headgroup. However, VacA bound SM and PC to approximately the same extent, suggesting that the phosphorylcholine head group common to both SM and PC may be an important determinant for VacA binding. We discuss below possible reasons why VacA binds to PC *in vitro*, while PC was not found to confer cellular sensitivity to VacA (Figure 1A and 1B).

### Lysenin Antagonizes VacA Interactions with SM

Finally, we investigated whether lysenin competitively inhibits VacA binding to SM-coated plates. Microtiter plates whose wells were coated with SM were incubated with VacA (10 nM) alone or mixed with different molar ratios of recombinant Venus-lysenin [47]. These experiments revealed that a molar excess of Venus-lysenin inhibited VacA binding to SM *in vitro* in a dose-dependent fashion (Figure 6C). In contrast, VacA cellular binding and vacuolation were only slightly inhibited (<20%) in the presence of a high molar excess (>1000-fold) of Venus-lysenin (data not shown), and we speculated that the inability to completely antagonize VacA binding may be explained in part by the high levels of SM within the plasma membrane. Notably, lysenin was not found to competitively inhibit VacA binding to PC *in vitro* (data not shown), which is consistent with previous reports that lysenin binds specifically to SM [32],[48].

## Discussion

By modulating important properties of eukaryotic cells, many toxins remodel the host environment to create a suitable niche for pathogenic organisms to colonize and persist during infection [14]. Many of the most potent toxins act upon intracellular targets. As the first step in cellular entry, intracellular-acting toxins bind to one or more plasma membrane surface receptors [7]. Notably, cells lacking toxin receptors are generally resistant to intoxication, underscoring the importance of the toxin-receptor complex [14].

Our results indicate that the important plasma membrane sphingolipid, SM, functions as a receptor for VacA. SM is the first plasma membrane component identified whose presence or absence is crucial for the sensitivity of epithelial cells to VacA, while at the same time dictating the extent to which the toxin binds to the cell surface. In contrast, although the plasma membrane proteins RPTP-α and RPTP-β confer cellular sensitivity to VacA and directly associate with the toxin *in vitro* [16]–[18], the significance of these proteins for VacA interactions with the cell surface has not been established. Likewise, while VacA has been reported to interact with several potential membrane components *in vitro*, including various lipid preparations, heparan sulfate, and glycosphingolipids, [19]–[23],[25],[49],[50], the significance of these interactions on the cell surface for VacA function remains to be experimentally addressed.

Our data indicate that VacA binds directly to SM *in vitro* (Figure 6). VacA also bound to PC *in vitro*, but not ceramide, suggesting that the phosphorylcholine head-group common to SM and PC may serve as a binding determinant for VacA. Surprisingly, incubation with exogenous PC did not increase the sensitivity of cells to VacA (Figure 1A, B). Moreover, exogenous dipalmitoyl-phosphatidylcholine (DPPC) or distearoyl-phosphatidylcholine (DSPC) did not increase VacA binding to HeLa cells (Figure S8). Depleting plasma membrane PC with phospholipase D type VII, which cleaves PC to produce phosphatidic acid and choline, did not decrease VacA binding to HeLa cells (data not shown). Neither SMase C nor SMase D pretreatment of monolayers detectably altered cellular PC levels (Table S3), indicating that decreased VacA cellular binding is unlikely to be due to contaminating PC-phospholipase C or phospholipase D activities within the SMase preparations used in these studies. Taken together, these data suggest that other differences between SM and PC not related to their common phosphorylcholine head group may be important for toxin vacuolation activity. Although SM and PC are both abundant in mammalian membranes [31], fundamental differences between the acyl chains of these two lipids result in SM partitioning predominantly to tightly packed, liquid-ordered phase domains (lipid-rafts), especially in the presence of cholesterol [30],[51], while PC is found predominantly in a liquid-disordered phase (non-rafts) [52]. Moreover, the molecular length of SM is significantly greater for SM than PC, and atomic force microscopy has revealed that SM protrudes out of the membrane, while PC is more embedded within the bulk membrane [52]–[54], which we speculate could result in PC being less accessible for VacA binding. Future work to evaluate the affinity of VacA for SM, as well as to define the structure-activity relationships of the lipid and the structure-function relationships of VacA important for this interaction, will be important for establishing the molecular basis and degree of VacA selectivity for this sphingolipid.

The propensity of SM to partition into lipid rafts may underlie, at least in part, the functional significance of this sphingolipid for VacA cellular intoxication. Several groups have demonstrated the importance of VacA association with lipid rafts for toxin-mediated cellular vacuolation [21], [27]–[29], but the molecular basis of raft association had been poorly understood previously. Here, we revealed that plasma membrane SM is critical for VacA association with lipid rafts (Figure 5). While it is not currently clear whether VacA binds directly to “pre-formed” raft domains, or whether VacA binding to SM induces the nucleation of new rafts, it is noteworthy that approximately 60% of total membrane SM has been estimated to partition normally into lipid rafts [55], and SM-rich membrane domains have been reported as a discrete subclass of lipid rafts [47],[48]. In considering a functional role for SM-rich rafts in VacA cellular intoxication, it is noteworthy that VacA association with lipid rafts precedes and is functionally important for the uptake of the toxin into cells [27],[29],[56]. In accordance, we found that HeLa cells depleted of plasma membrane SM internalized significantly less VacA compared to mock-treated cells (Figure S9). In contrast, CT and Tf were taken up to the same extent by SM-depleted and mock-treated cells (Figure S9C), suggesting that the cellular uptake pathways of these two proteins remained fully functional after depletion of plasma membrane SM.

How might the SM-dependent association of VacA with rafts promote toxin uptake? VacA is internalized into HeLa and AGS cells by a novel Cdc42-dependent, pinocytic-like mechanism, involving association of the toxin with rafts rich in glycosylphosphatidylinositol (GPI)-anchored proteins, although GPI-anchored proteins were not found to directly bind VacA [9], [11]–[13]. In considering potential roles for SM in VacA internalization, it is notable that Cdc42-dependent mechanisms of cell entry are regulated by levels of membrane sphingolipids such as SM, which are constantly recycled between the plasma membrane and several intracellular destinations [57]. One possibility, that remains to be determined, is that upon binding SM, VacA is taken up into cells by the same mechanism used for SM recycling from the cell surface [55].

A second possibility is that SM may function in concert with additional cell surface components, such as RPTP-β, to promote toxin entry into cells. It was recently reported that following the binding of VacA to AZ-521 cells, RPTP-β translocates from the [9] bulk membrane into lipid rafts [56], suggesting the interesting possibility that rafts might function as platforms where SM and RPTP-β function together to promote toxin uptake. However, additional raft components may also be important for toxin function as well. GPI-anchored proteins are enriched within lipid raft microdomains, but while one study indicated that enzymatic removal of these proteins decreased VacA function, there were no detectable effects on VacA interactions with DRMs [15]. Cholesterol is also enriched within lipid rafts, and facilitates SM packing within DRMs [58]–[60], and it is possible that cholesterol could function directly as a co-receptor for VacA cellular binding. However, we earlier reported that attempts to detect VacA interactions with cholesterol *in vitro* were unsuccessful [29]. In addition, several reports have indicated that extracting plasma membrane cholesterol with cyclodextrin compounds such as methyl-beta-cyclodextrin (MβCD) only partially reduces VacA association with DRMs and overall cell binding [27],[29],[56]. Although the membrane content of SM and cholesterol have been reported to be closely interdependent [61], we found treatment of cells with SMase D (100 μM) or SMase C (100 mU/mL) decreased plasma membrane content by only approximately 10% and 20% respectively (Figure S10). These same treatments decreased total cellular cholesterol levels by only approximately 10% and 9%, respectively, as compared to treatment with MβCD (4 mg/mL), which decreased total cellular cholesterol by approximately 60% (data not shown). Finally, exogenous cholesterol did not rescue VacA cellular vacuolation (Figure S4) or binding (Figure S7) in cells pretreated with SMase C. Based on these data, we suggest that rather than functioning as a VacA co-receptor, cholesterol may have an alternative function, such as contributing to the structural integrity of VacA-bound DRMs.

A third possibility is that SM is critical for the initial interaction of VacA with rafts on the cell surface, but does not participate directly in the internalization of the toxin. However, such a mechanism may not be likely, as VacA has been reported to remain associated with rafts subsequent to initial entry into cells [11].

Several studies have indicated that *H. pylori* produces a SMase *in vitro* [62],[63]. Currently it is unknown whether the *H. pylori* SMase is produced *in vivo* during infection, and if so, whether it is present at concentrations sufficient to deplete plasma membrane SM of gastric cells *in vivo*. Nonetheless, the possibility that *H. pylori* might generate both a SMase and SM-binding toxin *in vivo* suggests a potential post-translational mechanism by which *H. pylori* could regulate VacA activity during infection.

SM is an important structural component of lipid rafts within the cell membrane, while at the same time serving an important functional role in cell signaling, as it is the parent compound of several lipid mediators [30]. Here we provide the first example of a bacterial virulence factor that exploits this important membrane lipid as a cellular receptor. These results also provide a conceptual framework for future efforts to understand the molecular basis of toxin interactions with SM on the cell surface, and to develop inhibitors to block the action of VacA on sensitive host cells.

## Materials and Methods

### Purification of VacA

*H. pylori* 60190 (49503; ATCC), were cultured, and VacA was purified as described in ref. [64].

### Cell culture

HeLa (CCL-2; ATCC) and AZ-521 cells (3940; Japan Health Science Foundation) were maintained in minimum essential medium (MEM; Sigma). AGS cells (CRL-1739; ATCC) were grown in F-12K medium (ATCC). Tissue culture media were supplemented with 2 mM glutamine, 100 U penicillin/mL, 1 mg streptomycin sulfate/mL (Sigma), and 10% fetal bovine calf serum (FBS; JRH Biosciences), and maintained at 37 °C in a humidified atmosphere under 5% CO_2_.

### Preparation of recombinant sphingomyelinase D (SMase D) and Venus-lysenin

Recombinant SMase D [34] and Venus-lysenin [47] were expressed and purified as previously described in refs [65], and [47], respectively.

### Cell vacuolation

VacA was activated as described [66], and added to cells in the presence of 5 mM NH_4_Cl [67]. Cells were preincubated with the indicated concentrations of SM (860062; Avanti), phosphatidylcholine (PC, 840053; Avanti), dipalmitoyl-phosphatidylcholine (DPPC, 850355C; Avanti), or distearoyl-phosphatidylcholine (DSPC, 850365C; Avanti), phosphatidylethanolamine (PE, 840022; Avanti), phosphatidylinositol (PI, 840042; Avanti), or ceramide (ceram, 22244; Sigma) for 1 h prior at to intoxication at 37 °C. Cells were never exposed to greater than 1% solvent (methanol). In other experiments, monolayers were preincubated with the indicated concentrations of bacterial sphingomyelinase C (SMase C; Sigma), SMase D, or MβCD (Sigma), which alone did not cause a decrease in cell viability (as indicated by propidium iodide uptake). Cells were mock treated with the same volume of solvent or PBS as added with lipid or SMase C, SMase D, or MβCD respectively. To inhibit *de novo* SM synthesis, cells were treated with or without Fumonisin B_1_ (Sigma), which inhibits sphingosine N-acyltransferase [68], or (±)-threo-1-Phenyl-2-decanoylamino-3-morpholino-1-propanol (PDMP; Sigma), which inhibits SM synthase at concentrations≥25 mM [69]. Prior to administration of VacA, cells were incubated for 24 h with or without Fumonisin B_1_ (50 μg/mL) or PDMP (50 μM) in MEM (without FBS) +0.1% BSA.

Monolayers were visually examined using a Fisher Scientific MicroMaster inverted microscope, outfitted with a Nikon, Coolpix 43000 camera. For quantitative analysis, neutral red uptake was measured, as previously described [70]. Relative vacuolation was calculated by dividing neutral red uptake of lipid- or SMase-treated cells by neutral red uptake of cells incubated with VacA alone. For all experiments, neutral red uptake of mock-treated cells (e.g. minus VacA) was subtracted from the neutral red uptake of cells incubated with VacA.

### SM, PC, and cholesterol quantification

SM levels were measured using the Amplex Red Sphingomyelinase Assay Kit (Invitrogen/Molecular Probes; Eugene, OR), according to manufacturer's instructions [71]. Plasma membrane SM was visualized by incubating cells at 4 °C with Venus-lysenin, and then visualized by DIC/epifluorescence microscopy. PC levels were measured using the Amplex Red Phosphatidylcholine-Specific Phospholipase C Assay Kit (Invitrogen/Molecular Probes), according to manufacturer's instructions. Total cellular cholesterol levels were measured using the Amplex Red Cholesterol Estimation Kit (Invitrogen/Molecular Probes), according to manufacturer's instructions. Plasma membrane cholesterol was evaluated by staining cells with Filipin III (50 μg/ml; Sigma), and analyzing by flow cytometry using UV laser excitation, as previously described [72]. As a standard quality control measure, cellular SM, PC, or cholesterol levels were quantified for every experiment involving depletion or supplementation of cellular SM, PC, or cholesterol.

### Labeling VacA

Purified VacA was conjugated with Alexa Fluor 488, using the Alexa Fluor labeling kit (Invitrogen) according to the manufacturer's instructions. Labeling was experimentally determined to not alter VacA-mediated cellular vacuolation activity. Alexa Fluor 488 labeled-Tf and -CTB were from Invitrogen.

### DIC-epifluorescence Microscopy

Chamber slides were analyzed using a Delta Vision RT microscope (Applied Precision), EX 490/20 and EM 528/38, using an Olympus Plan Apo 60x oil objective with NA 1.42 and working distance of 0.17 mm. DIC images were collected for all fields. Images were processed using SoftWoRX Explorer Suite.

### Flow cytometry

Analytical flow cytometry was carried out using a Coulter EPICS XL-MCL™ flow cytometer equipped with a 70-μm nozzle, 488 nm line of an air-cooled argon-ion laser, and 400 mV output. The band pass filter used for detection of cell fluorescence was 525/10 nm. Cell analysis was standardized for scatter and fluorescence by using a suspension of fluorescent beads. Events were recorded on a log fluorescence scale and the geometric mean fluorescence values were determined using FCS Express 3.00.0311 V Lite Standalone. The data were gated so that only viable cells, which were readily evident by their forward and side scatter properties, were considered, while non-viable cells (which demonstrated lower forward scatter) were excluded from analysis.

### VacA binding and internalization

Experiments analyzed by DIC/epifluorescence microscopy were performed with monolayers of cells in 8-well chamber slides (Nunc), while experiments analyzed by flow cytometry were carried out with suspended cells (10^6^ cells/mL). As specified, cells were preincubated for 1 h in the presence of SM or SMase C. Cells pre-chilled to 4 °C were incubated for 1 h at 4 °C with activated Alexa Fluor 488-labeled VacA (10 or 100 nM), Alexa Fluor 488-labeled CTB (100 nM), Alexa Fluor 488-labeled Tf (60 nM), or Venus-lysenin (1 μM). For binding, cells were washed twice with PBS and fixed with paraformaldehyde (4%) on ice. For internalization, cells were washed once with ice-cold PBS 7.2, and then incubated at 37 °C in MEM plus 10% FBS (prewarmed to 37 °C). After 1 h, cells were incubated for 5 min on ice with trypan blue (0.5% in PBS 7.2), which is a membrane-impermeable, Alexa Fluor 488 fluorescence quenching agent [73],[74], and then analyzed immediately by flow cytometry. In preliminary experiments, we confirmed by microscopy that trypan blue quenched the fluorescence of membrane-bound, but not intracellular, Alexa Fluor 488-labeled VacA. Relative binding or internalization was calculated by dividing the geometric mean fluorescence of lipid- or SMase-treated cells by the geometric mean fluorescence of cells incubated with VacA alone. For all experiments, the geometric mean fluorescence of mock-treated cells (e.g. minus VacA) was subtracted from the geometric mean fluorescence of cells incubated with VacA.

### Preparation and analysis of DRMs

DRM preparation and analysis were performed as previously described [29]. DRMs were fractionated using OptiPrep (Sigma) density centrifugation, and proteins were detected by Western blot analysis using VacA rabbit antiserum (Rockland Immunochemicals), anti-cholera rabbit antiserum (Sigma), anti-Tf-R mouse antibodies (Zymed), anti-rabbit or anti-mouse immunoglobulin G-alkaline phosphatase conjugates (Sigma), and Lumi-phos substrate (Pierce).

### Enzyme-linked Immunosorbent Assay (ELISA)

VacA binding to lipids was assessed by ELISA, as described previously [32],[46], in lipid-coated Immulon 1B microtiter plates (Fisher). VacA binding was detected using VacA antiserum, followed by HRP conjugated anti rabbit antibodies (Rockland) and signal was detected using Ultra TMB-ELISA substrate (Pierce). Relative VacA binding was calculated by dividing the absorbance at 450 nm in the presence of lipid by that detected in the absence of lipid.

### Statistics

Unless otherwise indicated, each experiment was performed at least three independent times. For those studies requiring statistical analysis, data are from a representative experiment conducted in triplicate. All statistical analyses were performed using Microsoft Excel (Version 11.0). Error bars represent standard deviations. All *P* values were calculated with the Student's *t* test using paired, two-tailed distribution. Asterisks indicate statistical significance (*P*<0.05).

## Supporting Information

Figure S1 VacA-SM mixtures of with SM Potentiates VacA-Mediated Cellular Vacuolation. Monolayers of HeLa cells were pre-treated with SM (50 μM; labeled as “preincubated with SM prior to VacA incubation”) or mock treated with PBS pH 7.2 (labeled as “no lipid”) for 1 h at 37 °C and under 5% CO_2_, and then further incubated with activated VacA (10 nM). Alternatively, VacA (10 nM) was premixed with SM (50 μM; labeled as “SM-VacA mixture added to cells concurrently”) for 1 h at 25 °C, and then added to monolayers of HeLa cells. In each case, the HeLa cells were incubated in the presence of VacA at 37 °C for a total of 24 h, and then scored for vacuolation by measuring neutral red uptake. The data were normalized to the neutral red uptake in mock-pretreated cells incubated with VacA. Asterisks indicate statistically significant differences in neutral red uptake between mock-pretreated cells and cells either pretreated with SM or to which the SM/VacA mixture was added.
(359 KB EPS)

Figure S2 Effects of Exogenous SMase on Plasma Membrane SM. HeLa cells were treated with SMase C (100 mU/mL) or mock treated for 1 h at 37 °C, and then further incubated at 4 °C for 1 h with Venus-lysenin (1 μM). The cells were analyzed by epifluorescence- (panels 1 and 3) and DIC-microscopy (panels 2 and 4). The micrographs indicate the relative amounts of SM on the plasma membrane of representative cells pre-incubated with (panel 3) or without (panel 1) SMase C, as indicated by the amount of exogenously-added Venus-lysenin. The DIC images are provided to show the overall morphology of the cells presented in the accompanying fluorescence micrographs.
(2709 KB EPS)

Figure S3 The Dependence of VacA Activity on Plasma Membrane SM Occurs in Multiple Cell Lines. Monolayers of HeLa (black bars), AGS (shaded bars), or AZ-521 (white bars) cells (5×10^4^ cells/mL) were pre-incubated with SMase C (6.25 – 100 mU/mL) or mock-pretreated with PBS 7.2 for 1 h at 37 °C and under 5% CO_2_, and then further incubated with activated VacA (100 nM). After 24 h, the cells were scored for vacuolation by measuring neutral red uptake. For, each cell line, the data were normalized to the neutral red uptake of the mock-pretreated cells incubated with VacA. Asterisks indicate statistically significant differences in neutral red uptake between cells pretreated with SMase C and mock-pretreated cells.
(375 KB EPS)

Figure S4 Exogenous SM, but not Exogenous PC, PI, PE, or Cholesterol Rescues VacA Cellular Vacuolation in Monolayers Previously Depleted of SM by SMase C Treatment. Monolayers of HeLa cells were pretreated with SMase C (100 mU/mL) or mock-pretreated with PBS pH 7.2 (black) for 1 h at 37 °C and under 5% CO_2_. The pretreated cells were then washed, chilled to 4 °C, and incubated with SM (red), cholesterol (green), PC (blue), PE (orange) and PI (purple) (0–200 μM) or mock treated with PBS pH 7.2 (white) at 4°C for 1 h. Each of the monolayers was further incubated with activated VacA (100 nM) in the presence of exogenous lipids at 37 °C for 24 h, and then scored for vacuolation by measuring neutral red uptake. Vacuolation was normalized to neutral red uptake in mock-pretreated cells incubated with VacA. Asterisks indicate statistically significant differences in neutral red uptake between cells incubated with SMase C and then exogenous lipids, and cells incubated with SMase C and then PBS pH 7.2.
(387 KB EPS)

Figure S5 Ceramide Enrichment does not Inhibit VacA-Mediated Cellular Vacuolation. (A) Monolayers of HeLa cells were pretreated with ceramide (0–50 μM) or mock-pretreated with PBS pH 7.2 within a humidified environment at 37 °C under 5% CO_2_, and then incubated with activated VacA (100 nM). After 24 h, the cells were scored for vacuolation by measuring neutral red uptake. There were no significant differences in neutral red uptake between the ceramide pretreated and mock-pretreated cells. (B) HeLa cells were pretreated with SMase D (0–100 μM) or mock pretreated with PBS pH 7.2 within a humidified environment at 37 °C under 5% CO_2_. After 1 h, the cells were washed, and the cells were further incubated with activated VacA (100 nM), and scored for neutral red uptake after 24 h. The data were normalized to neutral red uptake measured in mock-pretreated cells incubated with VacA. Asterisks indicate statistically significant differences between cells pretreated with SMase D and mock-pretreated cells.
(370 KB EPS)

Figure S6 VacA Binding to Cells is Dependent on Cellular SM Levels. Monolayers of HeLa cells were pre-incubated with or without SMase C (0–50 mU/mL) at 37 °C and under 5% CO2. After 1 h, the cells were chilled to 0 °C on ice, and then incubated with Alexa Fluor 488-labeled VacA (50 nM) at 4 °C. After 1 h, the cells were analyzed for toxin binding and cellular SM content. For each SMase concentration used, we plotted the percentage of VacA binding (y-axis) and cellular SM concentration (x-axis) relative to control cells that had not been treated with SMase C. The R^2^ value was calculated by linear regression analysis of the experimental data. This experiment was performed two independent times. The data are from a representative experiment conducted in triplicate.
(371 KB EPS)

Figure S7 Exogenous SM, but not Exogenous PC, PI, PE, or Cholesterol Rescues VacA Cellular Binding in Monolayers Previously Depleted of SM by SMase C Treatment. Monolayers of HeLa cells were pre-treated with SMase C (100 mU/mL) or mock treated with PBS pH 7.2 (black) for 1 h at 37 °C and under 5% CO2. The pre-treated cells were then chilled to 4 °C, and incubated with SM (red), cholesterol (green), PC (blue), PE (orange) and PI (purple) (0–200 μM) or mock treated with PBS pH 7.2 (white) at 4°C for 1 h. Each of the monolayers was further incubated with Alexa Fluor 488 labeled VacA (50 nM) at 4 °C for 1 h, and then analyzed for VacA binding by monitoring Alexa Fluor 488 fluorescence using flow cytometry. Binding was normalized to the geometric mean fluorescence of mock-pretreated cells incubated with VacA. Asterisks indicate statistically significant differences in VacA binding between cells incubated with SMase C and then exogenous lipids, and cells incubated with SMase C and then PBS pH 7.2.
(385 KB EPS)

Figure S8 Exogenous DPPC / DSPC does not Increase VacA Binding to HeLa Cells. HeLa cells were pretreated with DPPC (200 μM), DSPC (200 μM), or mock-pretreated with PBS pH 7.2 at 4 °C for 1 h. The cells were then further incubated with Alexa Fluor 488-labeled VacA (10 nM) and exogenous lipids at 4 °C for 1 h. The cells were then analyzed for VacA binding by monitoring Alexa Fluor 488 fluorescence using flow cytometry. Binding was normalized to the geometric mean fluorescence of mock-pretreated cells incubated with VacA. Asterisks indicate statistically significant differences in VacA binding between cells preincubated with exogenous lipids and mock-pretreated cells.
(346 KB EPS)

Figure S9 VacA Entry into Cells is Dependent on Plasma Membrane SM. HeLa cells pretreated with SMase C (100 mU/mL) (A; panels 3 and 4) or mock-pretreated with PBS pH 7.2 (A; panels 1 and 2), and then further incubated at 37 °C with Alexa Fluor 488 labeled VacA (100 nM). For (C), cells were also incubated with Alexa Fluor 488 labeled cholera toxin B fragment (100 nM), or Alexa Fluor 488 labeled transferrin (60 nM). After 1 h, the cells were analyzed by DIC/epifluorescence microscopy (A) or flow cytometry (B, C) in the presence of trypan blue (0.5%), a membrane impermeable dye that quenches extracellular Alexa Fluor 488 fluorescence, thus allowing measurement of only intracellular Alexa Fluor 488-labeled VacA.
(A) Fluorescence micrographs (left-hand panels) and DIC images (right-hand panels) of VacA internalized within cells that had been pre-treated with (panels 3–4) or without SMase (panels 1–2).
(B) Flow cytometry histograms of Alexa Fluor 488 fluorescence of cells alone, cells incubated with VacA, and cells that had been pre-treated with SMase prior to exposure to VacA, as indicated.
(C) Relative internalization of VacA, CT, and Tf, as indicated, to cells that had been pre-incubated with (black bars) or without (white bars) SMase. Data were normalized for internalization of each protein in the mock-pretreated cells. For each protein, *P* values were calculated to determine statistically significant differences in binding between cells pretreated with SMase C and those mock-pretreated.
(3190 KB EPS)

Figure S10 Effects of SMase C or SMase D on the Plasma Membrane Cholesterol Content. Monolayers of HeLa cells were treated with SMase C (100 mU/mL), SMase D (100 μM), MβCD (4 mg/mL), or mock treated with PBS pH 7.2 for 1 h at 37 °C. The cells were fixed by incubation with paraformaldehyde (2%) for 1 h at 25 °C, stained with Filipin III (50 μg/ml; 4 h), and evaluated by flow cytometry. The data were normalized to Filipin III staining of mock-treated cells. Asterisks indicate statistically significant differences between cells pretreated with SMase C, SMase D, or MβCD, and mock-treated cells.
(354 KB EPS)

Table S1 Effects of Exogenous SM on Cellular SM Content. SM content was quantified in HeLa cells that had been incubated with or without exogenous SM. SM was quantified as a function of exogenous SM concentration, time, and temperature.
(450 KB EPS)

Table S2 Effects of Exogenous SMase C/D on Cellular SM Content. SM content was quantified in HeLa cells that had been incubated with or without SMase C. SM was quantified as a function of SMase C concentration, time, and temperature.
(407 KB EPS)

Table S3 Effects of Exogenous SMase C/D on Cellular PC Content. PC content was quantified in HeLa cells that had been incubated with or without SMase C.
(391 KB EPS)
